# Influence of Heat Treatment on the Phase Composition of Biphasic Calcium Phosphates for Biomedical Application

**DOI:** 10.7759/cureus.83514

**Published:** 2025-05-05

**Authors:** Tsanka Dikova, Ivaylo Parushev, Vladimir Dunchev, Ralitsa Yotsova, Ismail Ismailov, Ivaylo Ivanov, Hasan Hasanov

**Affiliations:** 1 Department of Dental Material Science and Prosthetic Dental Medicine, Faculty of Dental Medicine, Medical University of Varna, Varna, BGR; 2 Department of Periodontology and Dental Implantology, Faculty of Dental Medicine, Medical University of Varna, Varna, BGR; 3 Department of Material Science and Mechanics of Materials, Faculty of Mechanical and Precision Engineering, Technical University of Gabrovo, Gabrovo, BGR; 4 Department of Oral Surgery, Faculty of Dental Medicine, Medical University of Varna, Varna, BGR; 5 Department of Chemistry, Faculty of Natural Sciences, Konstantin Preslavsky University of Shumen, Shumen, BGR

**Keywords:** biomedical application, biphasic calcium phosphates, heat treatment parameters, phase ratio, wet precipitation

## Abstract

The aim of the present paper is to study the influence of the heat treatment on the phase composition of biphasic calcium phosphates (BCPs) for biomedical applications. The precursors were prepared by the wet precipitation method using calcium nitrate tetrahydrate and diammonium hydrogen phosphate. The as-synthesized powder was subjected to heat treatment at different temperatures and times. Qualitative and quantitative analyses of the phase composition were done by X-ray diffraction (XRD) and Fourier transform infrared spectroscopy (FTIR). A comparative analysis of the phase composition of the obtained BCPs was performed. It was found that the as-synthesized precursor was characterized by a biphasic brushite/monetite structure. The heat treatment at 700°C caused the formation of a triphasic structure of monetite, β-calcium pyrophosphate (β-CPP), and β-tricalcium phosphate (β-TCP). Heat treatment in the temperature range of 900°C-1300°C led to a change in the precursor’s phases. The structure of the heat-treated powders was biphasic, consisting of β-CPP as the main phase and β-TCP. Increasing the calcination temperature led to an increase in the β-TCP amount from 23.5 % at 900°C up to 36.5 % at 1300°C and a decrease in the β-CPP amount from 76.5 % to 63.5 %, respectively. The holding time in heat treatment did not strongly influence the phase ratio. Increasing the duration leads to a variation in the β-CPP/β-TCP ratio within a very tight range: 76.8/23.2% to 73.4/26.6%. The new data on the influence of heat treatment on the β-CPP/β-TCP ratio can be used for optimizing process parameters in the design of new BCPs for biomedical applications.

## Introduction

Calcium phosphate ceramics (CPCs) have long been recognized as biomaterials for bone regeneration with excellent biological properties, such as biocompatibility, osteoconductivity, and nontoxicity. These properties, along with their cost-effectiveness and relatively easy manufacturing, make them preferred and suitable materials in bone reconstructive surgery [1-3].

The biphasic calcium phosphates (BCPs) were first introduced at the end of the 19^th^ century [2]. Since then, they have been widely used in general and dental medicine for bone substitution and scaffolding, implant coating, and drug delivery due to their controlled bioactivity and solubility [1-4]. The main components of BCPs are hydroxyapatite (HA) and β-tricalcium phosphate (β-TCP). The material resorption and its substitution with bone depend on the HA/β-TCP ratio [5, 6]. HA and β-TCP have different calcium-to-phosphorus (Ca/P) ratios of 1.67 and 1.5, respectively, which can affect their mechanical and biological properties [7-9]. HA has comparatively higher mechanical properties and a low degradation rate. That is why it serves as a scaffold for bone ingrowth. On the other hand, TCPs are resorbed more rapidly and have lower mechanical properties. There are two modifications of TCPs: α-TCP and β-TCP, which have the same composition but different crystalline structures and lower biodegradability (α-TCP > β-TCP > HA). An inverse relationship has been found between the material’s solubility and the Ca/P ratio in the interval of 1-1.67 [1, 2, 10]. The HA/β-TCP combination enables improved resorbability and enhanced mechanical properties of BCPs, achieved by adjusting the Ca/P ratio [2, 3].

Various thermal methods have been employed to obtain BCPs, including high-temperature sintering, solid-state synthesis, combustion, and flame spray pyrolysis [2, 11, 12]. Other methods include sol-gel or wet precipitation reactions followed by heat treatment [13-15]. The production methods can influence the structure and medical properties of the obtained BCPs.

Wet precipitation synthesis is a simple and cost-effective method for the preparation of BCP precursors [16-19]. It is carried out through a chemical reaction in solution, which occurs with the formation of a precipitate. In the next stage, the dried precipitate is subjected to heat treatment (calcination), during which phase transformations occur and the final products are obtained. The phase composition of the as-obtained BCPs depends on the wet precipitation and heat treatment parameters, such as initial materials (Ca/P ratio), solution pH, and stirring speed on one hand and calcination temperature and duration on the other [20-24]. Any variation in these parameters can alter the final product.

It is stated by Massit et al. [20] that the most conventional method for the synthesis of precursors for β-TCP is the precipitation in an aqueous medium from calcium nitrate tetrahydrate and diammonium hydrogen phosphate as starting materials. However, this method is very sensitive and needs close control of all parameters. Depending on the parameter variations, a mono- and biphasic structure, composed of β-TCP with pyrophosphates or HA, resulted. By varying the synthesis or heat treatment parameters, Xidaki et al. have prepared single β-TCP or HA and biphasic HA/β-TCP powders with different content [9]. Kwon et al. have described a synthesis of β-TCP and biphasic HA/β-TCP through a coprecipitation method using the same starting materials [25]. It is found that the starting Ca/P ratio and the solution pH influence the formation of different phases, as the initial Ca/P ratio could control the HA content of the biphasic powder. In their investigation, Mirhadi et al. have used calcium nitrate and diammonium hydrogen phosphate as precursors for the synthesis of nano-sized β-TCP and HA/β-TCP composite [24]. They have confirmed that the Ca/P ratio increases up to 1.59 when increasing the pH up to 10.8, thus changing the phase composition of the obtained powders. Topsakal et al. have established the values of the solution pH and calcination temperature for obtaining single, biphasic, or even triphasic CPCs [18]. However, unlike other authors, the biphasic powders are composed of β-TCP and β-calcium pyrophosphate (β-CPP) at pH=10 or 11 and temperature above 900°C, while at a lower temperature of 700°C, a triphasic HA/β-CPP/α-CPP structure results. After two hours of sintering at 700°C, Sych et al. have obtained polyphosphate ceramics, consisting of HA, β-CPP, and β-TCP phases, as their amount depends on the initial HA/monetite ratio [26, 27].

The abovementioned examples are evidence that any variations in the parameters of wet precipitation and heat treatment processes change the final product. Thus, the wet-chemical reaction and high-temperature calcination for the preparation of BCPs often lead to the obtaining of different final products [9, 18, 24, 25]. Further research is necessary to define the optimal parameters of wet precipitation and heat treatment for obtaining BCPs with the desired composition.

The present paper aims to study the influence of the heat treatment parameters (temperature and holding time) on the phase composition of BCPs for biomedical applications. The precursor is prepared by the wet precipitation method using calcium nitrate tetrahydrate and diammonium hydrogen phosphate. The as-synthesized powder is subjected to heat treatment at different temperatures and times. A comparative analysis of the phase composition of the obtained BCPs is performed.

## Materials and methods

Wet precipitation synthesis

For preparing BCPs in the present study, calcium nitrate tetrahydrate and diammonium hydrogen phosphate were used to ensure an initial Ca/P ratio of 1.5. At first, 150 ml of 0.6 mol solution of calcium nitrate tetrahydrate (Chempur, Piekary Slaskie, Poland) was added slowly (2 ml/min) dropwise to 150 ml of 0.4 mol solution of diammonium hydrogen phosphate (Chempur, Piekary Slaskie, Poland). The pH of the obtained solution was corrected to nine by adding ammonium hydroxide solution (25% ammonium hydroxide, Chempur). After that, it was stirred at 600 rpm speed by magnetic stirrer (MS10-H500-Pro, DLAB Scientific Co., Ltd., Beijing, China) for 24 hours at room temperature, and the resulting suspension was filtered. The obtained precipitate was washed twice with distilled water and absolute ethyl alcohol and then dried for 24 hours at 120°C in a laboratory dryer (DZ-2BL, Faithful Instrument Co., Huanghua, China). The dried precipitate was ground in a mortar and divided into portions of 2 grams each, which were subjected to heat treatment.

Heat treatment

The as-synthesized powders were subjected to heat treatment (calcination) in a furnace LH 30/14 (Nabertherm GmbH, Lilienthal, Germany) at different regimes, varying with the temperature (700°C-1300°C) and holding time (one to four hours). The designation of the samples and the calcination parameters are given in Table 1.

**Table 1:** Designation, calcination parameters, and phase composition of the samples

№	Probe designation	Calcination		Phase composition	
		Temperature, °C	Time, h	Crystalline phase, %	Amorphous phase, %
1	M2-0-0	0	0	74.9	25.1
2	M2-700-1	700	1	66.5	33.5
3	M2-700-4	700	4	70.5	29.5
4	M2-900-2	900	2	76.7	23.3
5	M2-1000-1	1000	1	75.2	24.8
6	M2-1000-2	1000	2	74.6	25.4
7	M2-1000-4	1000	4	79.8	20.2
8	M2-1100-2	1100	2	84	16
9	M2-1200-2	1200	2	86.5	13.5
10	M2-1300-2	1300	2	82.8	17.2

Samples characterization

The chemical and phase composition of the as-synthesized and heat-treated powders was investigated by X-ray diffraction (XRD) and Fourier transform infrared spectroscopy (FTIR) analysis. The XRD analysis was performed in Cu Kα irradiation in the range of 2Θ=9-54° with a step of 0.02° using a Bruker D8 Advance diffractometer (Bruker AXS GmbH, Karlsruhe, Germany). A specialized software, DIFFRAC.DQuant V1.5, developed by Bruker, was used for the identification of the individual phases and their relative ratio. The codes and the designation of the obtained phases are shown in Table 2. The bonding architecture and type of functional groups in the samples were investigated by FTIR analysis in the range of 4000-500 cm⁻¹ using Shimadzu BX II (Shimadzu Corporation, Kyoto, Japan).

**Table 2:** Crystalline phases in the investigated probes XRD - X-ray diffraction

XRD data		Chemical formula	Name	Abbreviation
COD	Calculated formula			
2106184	CaHPO4	CaHPO_4_	Dicalcium phosphate anhydrous (monetite)	DCPA
1533075	CaH5O6P	CaHPO_4_.2H_2_O	Dicalcium phosphate dihydrate (brushite)	DCPD
1517238	Ca3O8P2	Ca_3_(PO_4_)_2_	Beta-tricalcium phosphate	β-TCP
1001556	Ca2O7P2	Ca_2_P_2_O_7_	Beta-calcium pyrophosphate	β-CPP

**Figure 1:**
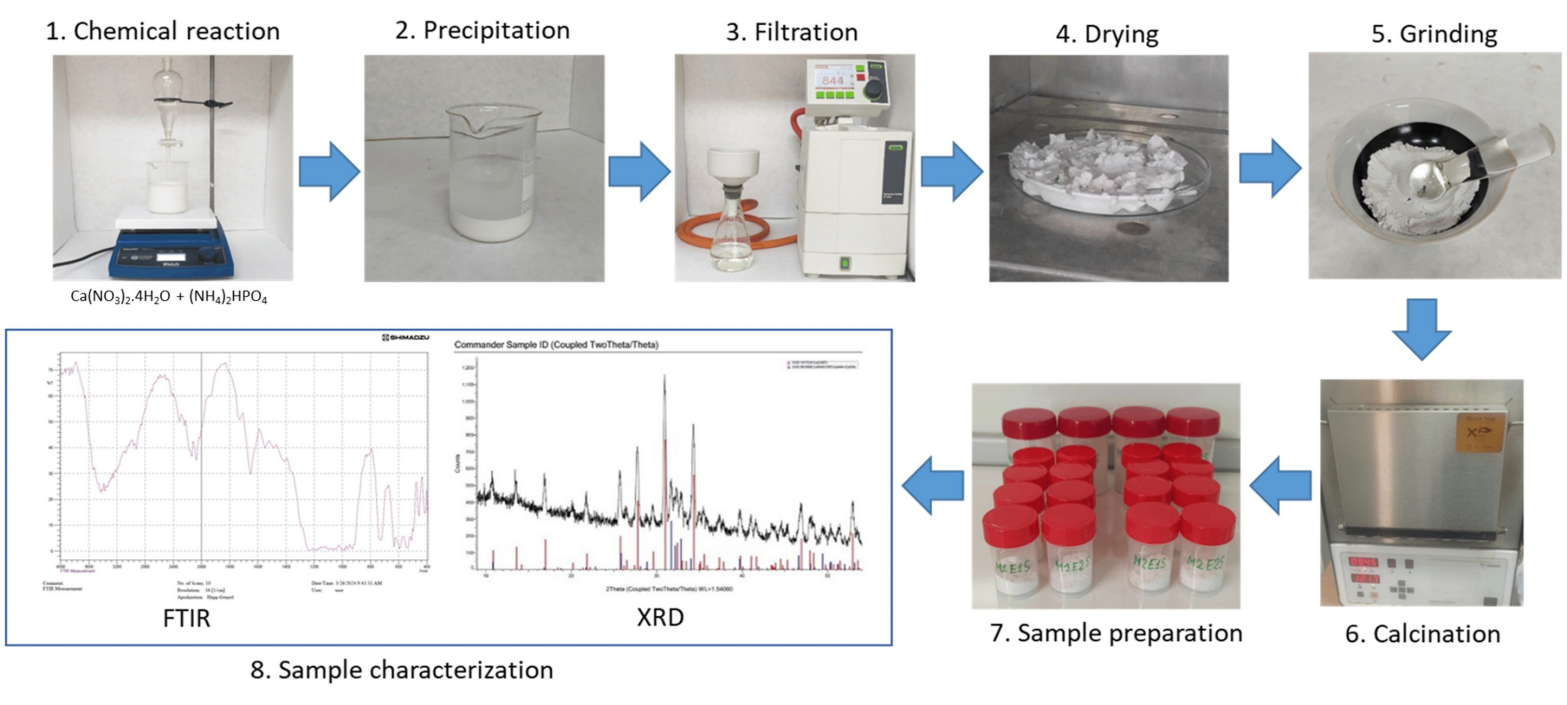
Flow chart of the study protocol FTIR: Fourier transform infrared spectroscopy; XRD: X-ray diffraction

The steps of the protocol are presented in Figure 1.

## Results

Phase composition

The synthesized powders before and after heat treatment consisted of crystalline and amorphous phases in different ratios, which depended on the calcination parameters (Table 1). The ratio between the crystalline and amorphous phases in the as-synthesized powder was 74.9%/25.1%. In the temperature range of 900°C to 1300°C, the crystalline phase amount increased with the temperature increase and reached a maximum of 86.5% at 1200°C.

**Figure 2:**
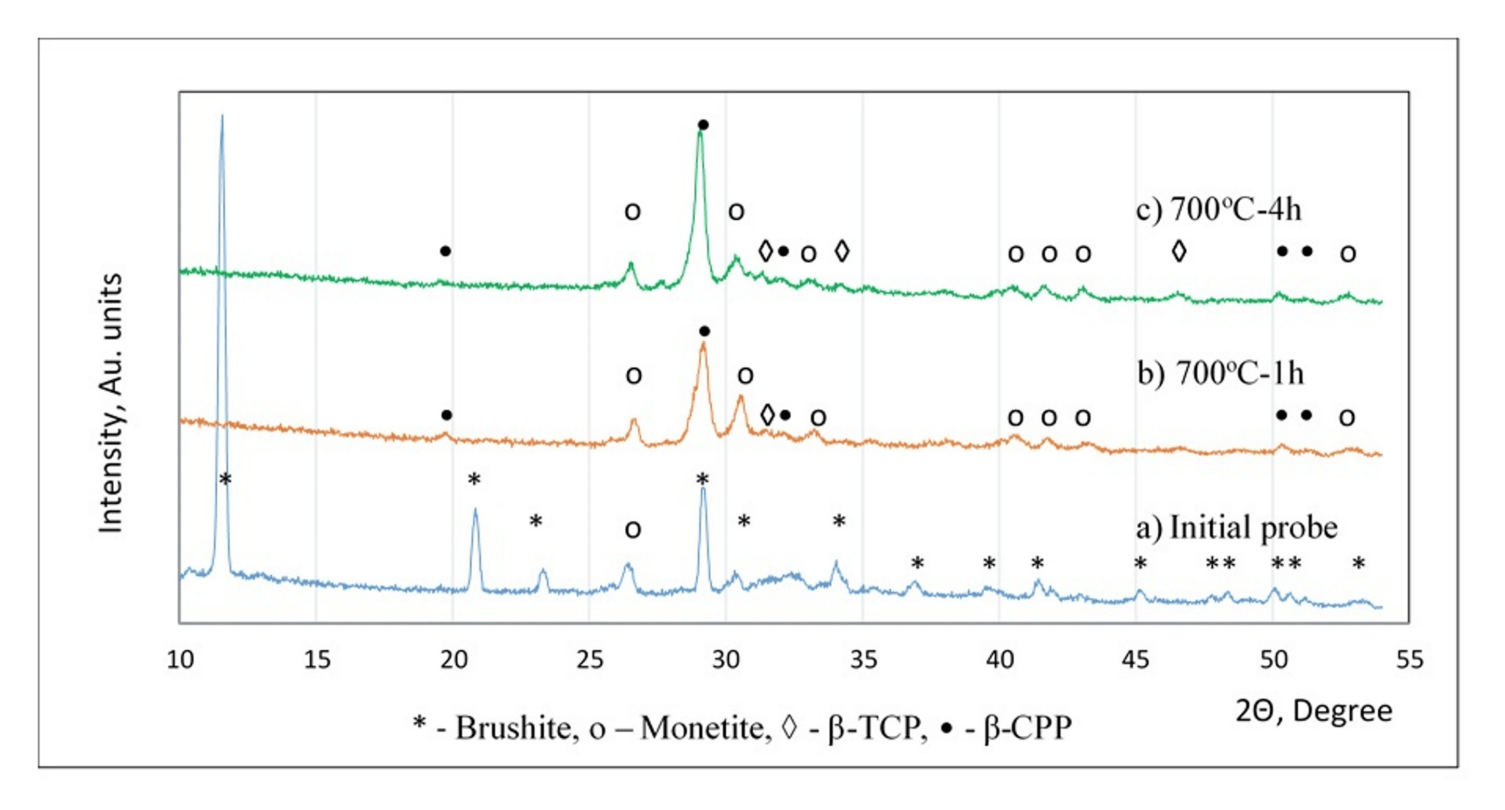
Phase composition of the synthesized powder Diffractograms of powders before and after calcination: a) initial probe; b) 700^o^C, one hour (h); c) 700^o^C, four hours; X-ray diffraction (XRD) peaks: *: brushite; o: monetite; ◊: β-tricalcium phosphate (β-TCP); •: β-calcium pyrophosphate

The diffractograms in Figure 2 show the phase composition of the synthesized powder before and after calcination at 700°C. Clear and sharp peaks, mainly of brushite and some low peaks of monetite, were observed in the as-synthesized probe. As monetite is stable at temperatures above 100°C [3], maybe the presence of monetite was due to dehydration of brushite during 24 hours of drying at 120°C after synthesis. The calcination at 700°C led to a change in the phase composition. After a one-hour process, the peaks of brushite disappeared, and new peaks of monetite appeared. New phases were formed: the main phase β-CPP with the highest broad peak and a single peak of β-TCP with small intensity. Increasing the calcination time until four hours led to a sharp peak with a higher intensity of the β-CPP, decreasing the intensity of the monetite peaks, and the appearance of more β-TCP peaks.

**Figure 3:**
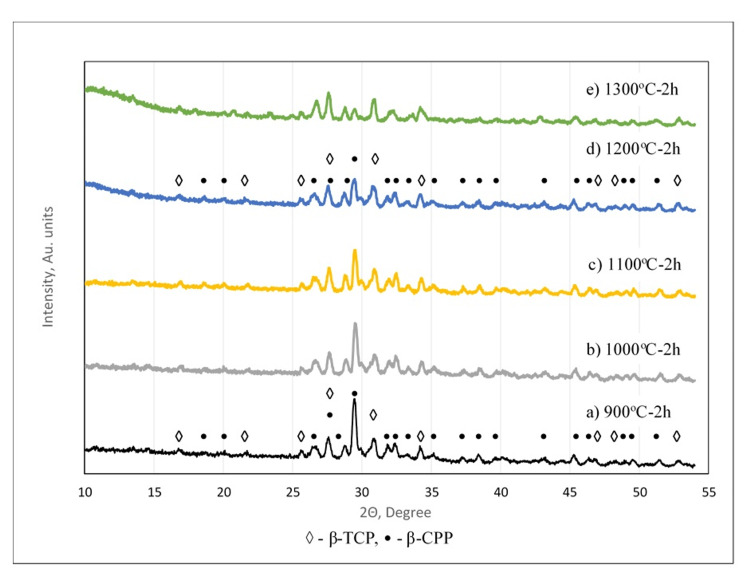
Phase composition of the synthesized powder after two hours (h) calcination at different temperature. Diffractograms of powders after two hours of calcination at a temperature of a) 900 ^o^C; b) 1000 ^o^C; c) 1100 ^o^C; d) 1200 ^o^C; e) 1300 ^o^C; X-ray diffraction (XRD) peaks: ◊: β-tricalcium phosphate (β-TCP); •: β-calcium pyrophosphate

Figure 3 shows the phase composition of the synthesized powders after two hours of calcination in the temperature range of 900°C-1300°C. Peaks of the β-CPP and β-TCP phases were observed in the diffractograms. Increasing the temperature led to a gradual decrease of the highest peak of the pyrophosphates and a slow increase of the β-TCP peak above 1200°C. Characteristic peaks of α-TCP were not present in the diffractograms at high temperatures (1200°C and 1300 °C).

**Figure 4:**
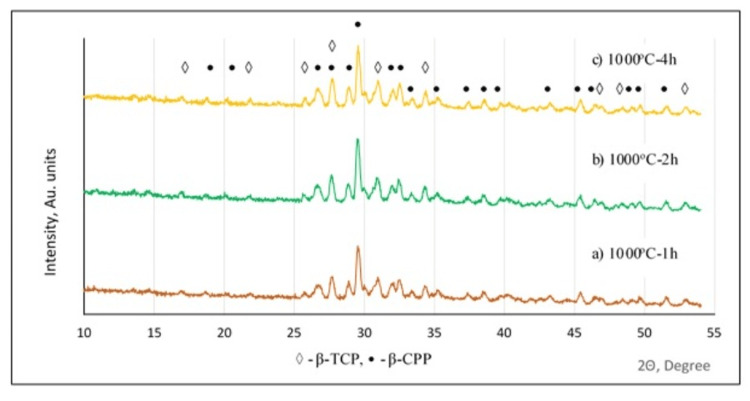
Phase composition of the synthesized powder after calcination Diffractograms of powders heat-treated at 1000°C and a holding time of: a) one hour (h) (h); b) two hours; c) four hours. X-ray diffraction (XRD) peaks: ◊: β-tricalcium phosphate (β-TCP); •: β-calcium pyrophosphate

Figure 4 shows diffractograms of powders heat-treated at 1000 °C and different holding times. Only peaks of pyrophosphates and β-TCP were observed, which did not change their intensity with increasing time from one to four hours. Therefore, the heat treatment led to a change of the phase composition from brushite and monetite of the as-synthesized sample to β-CPP and β-TCP of the heat-treated ones.

FTIR analysis

**Figure 5:**
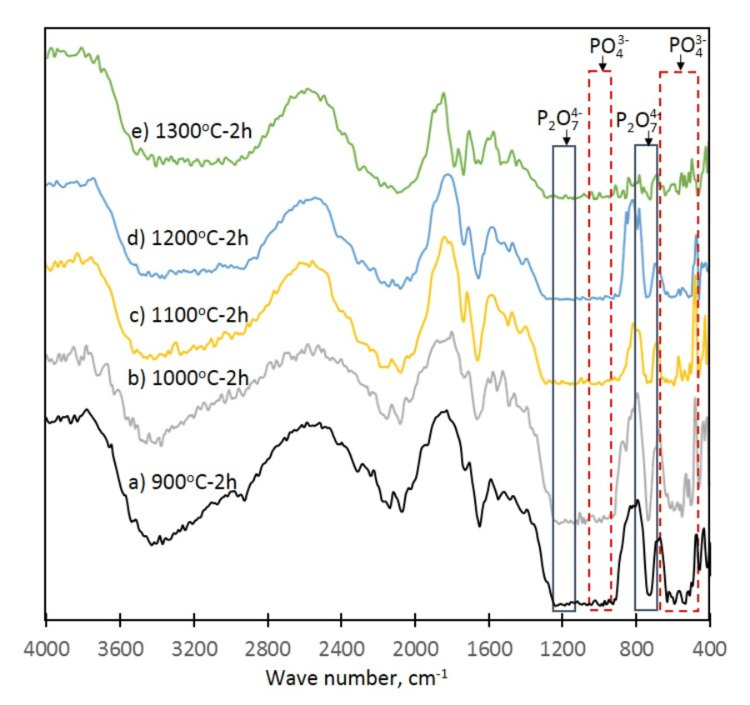
FTIR spectra of the synthesized powder after 2 hours (h) calcination at different temperatures Spectra of the synthesized powder after 2 h calcination at a temperature of: a) 900 ^o^C; b) 1000 ^o^C; c) 1100 ^o^C; d) 1200 ^o^C; e) 1300 ^o^C. FTIR: Fourier transform infrared spectroscopy; PO₄^3-^: phosphate group; P_2_O_7_^4-^: pyrophosphate group

The results of the FTIR investigation are shown in Figure 5 and Tables 3, 4. In the as-synthesized powder, the bands of the phosphate group (PO₄³⁻) at 532, 949, 1033, and 1157 cm⁻¹ were observed as well as bands of the (HPO₄)₂⁻ group at 586 cm⁻¹, 609 cm⁻¹, and 879 cm⁻¹, which can be attributed to the monetite and brushite. At the investigated temperature range of calcination (900-1300 °C), the peaks at 532 cm⁻¹-987 cm⁻¹ and 1018 cm⁻¹-1134 cm⁻¹ were typical for the phosphate group in the β-TCP. The characteristic peaks of the P2O74 group in the pyrophosphates were slightly shifted to 732 cm⁻¹-756 cm⁻¹ and 1188 cm⁻¹-1211 cm⁻¹.

**Table 3:** Characteristic FTIR peaks and corresponding functional groups present in the samples heat treated at 700 degrees Celsius FTIR: Fourier transform infrared spectroscopy; PO₄^3-^: phosphate group; P_2_O_7_^4-^: pyrophosphate group; β-CPP: β-calcium pyrophosphate; β-TCP: β-calcium pyrophosphate

Phases	β-TCP and monetite					β-CPP	
Functional groups	PO₄^3-^					P_2_O_7_^4-^	
Sample M2-700-1	540	555	979	1057	1141	725	1219
Sample M2-700-4	532	586	964	1080	1126	725	1211

**Table 4:** Characteristic FTIR peaks and corresponding functional groups present in the samples heat treated in the temperature range of 900-1300 degrees Celsius FTIR: Fourier transform infrared spectroscopy; PO₄3-: phosphate group; P2O74-: pyrophosphate group; β-CPP: β-calcium pyrophosphate; β-TCP: β-calcium pyrophosphate

Phases	β-TCP					β-CPP	
Functional groups	PO₄^3-^					P_2_O_7_^4-^	
Sample M2-900-2	532	594	964	1064	1095	725	1188
Sample M2-1000-1	540	594	941	1064	1111	725	1203
Sample M2-1000-2	545	594	987	1072	1111	732	1211
Sample M2-1000-4	555	578	933	1064	1111	732	1211
Sample M2-1100-2	555	586	933	1033	1126	725	1211
Sample M2-1200-2	555	586	979	1049	1134	740	1211
Sample M2-1300-2	555	632	972	1018	1134	756	1188

Comparative analysis

**Figure 6:**
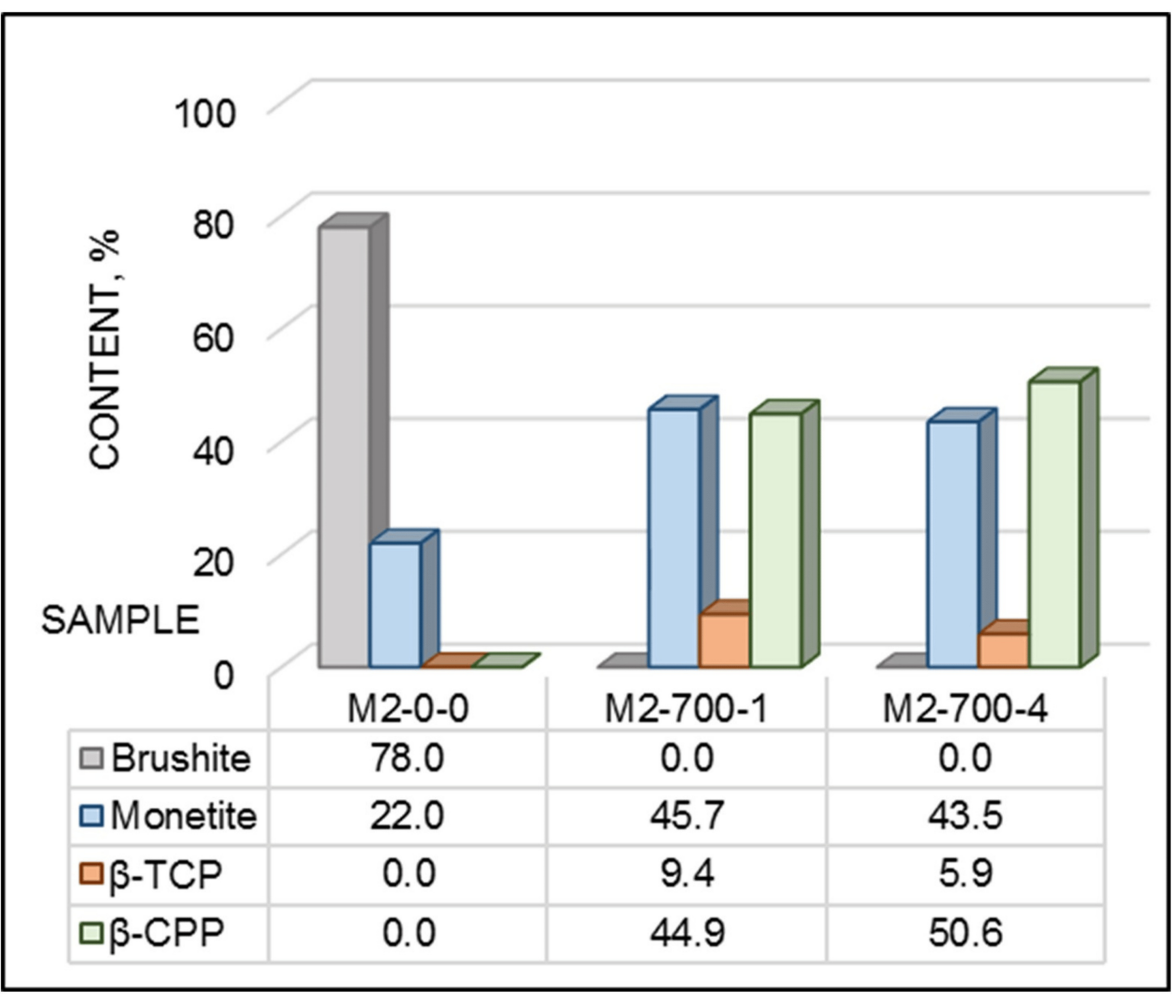
Phase ratio of the synthesized powder M2-0-0: before calcination; M2-700-1: after calcination at 700^o^C for one hour; M2-700-4: after calcination at 700^o^C for four hours; β-CPP: beta-calcium pyrophosphate; β-TCP: beta-tricalcium phosphate

**Figure 7:**
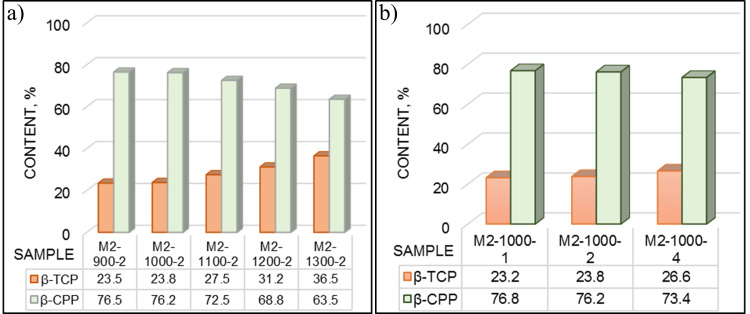
Influence of temperature: a) holding time; b) on the phase composition of the synthesized powder β-CPP: beta-calcium pyrophosphate; β-TCP: beta-tricalcium phosphate

Figure 6 and Figure 7 show the relative phase ratio of the as-synthesized powder and after heat treatment. The phase composition of the initial sample was biphasic and consisted of 78.0 % brushite and 22.0 % monetite (Figure 6). Calcination at 700°C led to the formation of a triphasic structure, consisting of monetite, pyrophosphates, and β-TCP. At the one-hour process, the powder was composed of 45.7% monetite, nearly the same amount of β-CPP (44.9%), and a small amount of β-TCP (9.4%). Increasing the holding time to four hours did not strongly influence the amount of the newly formed phases.

The structure of the powders, heat-treated in the high-temperature range of 900°C-1300°C, was biphasic, consisting of a higher quantity of β-CPP and a lower amount of β-TCP (Figure 7). Increasing the calcination temperature led to an increase of the β-TCP amount from 23.5 % at 900°C up to 36.5 % at 1300°C and a decrease of the β-CPP amount from 76.5 % to 63.5%, respectively (Figure 7a). The calcination duration did not strongly influence the phase ratio (Figure 7b). Only a low increase, with about 3% of the β-TCP amount at four hours of calcination, was observed. Increasing the duration led to variation of the β-CPP/β-TCP ratio in a very tight range from 76.8/23.2 % at one hour to 73.4/26.6 % at four hours of process.

## Discussion

In our study, calcium nitrate tetrahydrate and diammonium hydrogen phosphate are used for the synthesis of the precursor, which is characterized by a biphasic structure consisting of 78.0 % brushite and 22.0 % monetite (Figure 2 and Figure 6). As our solution was stirred for 24 hours, its pH reached a lower value, leading to the formation of a noticeable quantity of brushite. Part of the brushite was transformed into monetite during the following 24 hours of drying at 120°C.

The heat treatment at 700°C led to the formation of a triphasic structure of monetite/pyrophosphate/β-TCP (Figure 2 and Figure 6). It was found that at higher temperatures, the monetite transforms into CPP (Ca₂P₂O₇), as first γ-CPP is formed, which is then converted into β-CPP [22, 26, 27]. At higher temperature ranges, 600°C-1050°C, the interaction of the obtained phases ran, resulting in the formation of β-TCP [22, 28].

In the heat treatment at 900°C-1300°C, the initial biphasic structure was transformed into β-CPP and β-TCP, with the highest β-TCP of 36.5% in two hours of calcination at 1300°C (Figure 7). According to Topsakal et al. [18], using the same starting materials, biphasic structures, consisting of β-TCP and β-CPP, result in a higher pH of 10/11 and calcination above 900°C. Massit et al. [20] have found that the biphasic structure of β-TCP and β-CPP is obtained at a slow dropping rate of 3 ml/min, pH=9, and calcination at 800°C for one hour. As the parameters of the synthesis and following heat treatment are close to those in the works of Topsakal et al. [18] and Massit et al. [20], our results correspond well with their findings.

When calcium nitrate tetrahydrate and diammonium hydrogen phosphate are used as starting materials, mono- and biphasic structures composed of β-TCP with pyrophosphates or HA, the results depend on the variations of the synthesis parameters [16]. The biphasic structure, consisting of β-TCP and pyrophosphates, was obtained from a precursor synthesized at a reaction temperature of 30°C, a lower calcium nitrate tetrahydrate addition rate of 3 ml/min, an aging time of 72 hours, and pH=7, subjected to one hour of calcination at 800°C. Topsakal et al. [18] also have found a dependence of the phase composition on the environment's pH and calcination temperature. However, according to them, HA and pyrophosphates are prepared at pH=10 or 11 and calcination at 700°C. Increasing the calcination temperature above 900°C at the same pH led to the formation of a biphasic structure of β-TCP and β-CPP. Kwon et al. [25] have found β-TCP and β-CPP structures after calcination above 800°C of precipitates obtained at pH=8 with an initial ratio of Ca/P ≤1.0 (resulting ratio of Ca/P <1.5). It is stated in the work of Brazete et al. [23] that a small amount of HA is formed when Ca/P>1.5, while in Ca/P<1.5, a small amount of CPP appears. The presence of β-CPP delays the transition of β→α-TCP by increasing the transition temperatures on one hand. On the other hand, β-CPP facilitates the reversal α→β-TCP transformation.

The biphasic structure with β-CPP as the main component resulted after calcination at 900°C-1000°C. The β-TCP amount was low (23.5%-23.8 %). This may be due to the lack of HA, which could retard the formation of β-TCP [22, 28]. The high amount of pyrophosphates (76.5-63.5 %) could affect the β→α-TCP transformation, and as a result, no α-TCP is observed in our study.

In summary, it was found in the present study that the as-synthesized precursor was characterized by a biphasic brushite/monetite structure. The heat treatment at 700°C led to the formation of a triphasic structure of monetite/pyrophosphate/β-TCP. The heat treatment in the temperature range of 900°C-1300°C led to a change in the phases in the as-synthesized precursor. The structure of the heat-treated powders was biphasic, consisting of β-CPP as the main phase and β-TCP. Increasing the calcination temperature led to an increase in the β-TCP amount from 23.5% at 900°C up to 36.5% at 1300°C and a decrease in the β-CPP amount from 76.5% to 63.5%, respectively. The holding time in heat treatment did not strongly influence the phase ratio. Increasing the duration led to variation of the β-CPP/β-TCP ratio in a very tight range of 76.8%/23.2% and 73.4%/26.6%. The results obtained can be used to design new biofunctional materials with optimized resorbability and mechanical properties required for bone grafts and tissue engineering, where host responses to biomaterials can critically influence outcomes [29].

The BCPs in the present study were prepared by the wet precipitation method followed by calcination, as only the heat treatment parameters were investigated. Therefore, the limitations of the work include the influence of the synthesis process parameters, such as the type and concentration of the initial materials, the Ca/P ratio, the solution pH and temperature, and the stirring speed. All these factors are subjects of our future research. Optimizing the parameters of the chemical reaction will allow for the production of specific precursors. This, in turn, will allow for the formation of biphasic calcium phosphates with controlled phase composition through properly selected parameters of the thermal treatment.

## Conclusions

The influence of heat treatment on the phase composition of BCPs for biomedical applications is investigated in the present study. The precursor is prepared by the wet precipitation method using calcium nitrate tetrahydrate and diammonium hydrogen phosphate. The as-synthesized powder is subjected to heat treatment at different temperatures and times. Systematic experiments of heat treatment are conducted to investigate the phase transformations in the precursors. With these innovations, the study contributes to expanding the knowledge on the controlled synthesis of BCPs, which are essential for modern biomedical science.

## References

[1] Dorozhkin SV (2022). Calcium orthophosphate (CaPO4)-based bioceramics: preparation, properties, and applications. Coatings.

[2] Dorozhkin SV (2016). Multiphasic calcium orthophosphate (CaPO4) bioceramics and their biomedical applications. Ceram Int.

[3] Lobo SE, Arinzeh TL (2010). Biphasic calcium phosphate ceramics for bone regeneration and tissue engineering applications. Materials (Basel).

[4] Ebrahimi M, Pripatnanont P, Monmaturapoj N, Suttapreyasri S (2012). Fabrication and characterization of novel nano hydroxyapatite/β-tricalcium phosphate scaffolds in three different composition ratios. J Biomed Mater Res A.

[5] Jensen SS, Bornstein MM, Dard M, Bosshardt DD, Buser D (2009). Comparative study of biphasic calcium phosphates with different HA/TCP ratios in mandibular bone defects. A long-term histomorphometric study in minipigs. J Biomed Mater Res B Appl Biomater.

[6] Khayrutdinova DR, Goldberg MA, Antonova OS (2023). Effects of heat treatment on phase formation in cytocompatible sulphate-containing tricalcium phosphate materials. Minerals.

[7] Herdianto N, Gustiono D, Tasomara R (2021). Synthesis and characterization of mesoporous β-tricalcium phosphate. Mater Sci Forum.

[8] Wahjuningrum DA, Setiawan F, Utomo DN, Chusnita R, Syahrimayani A, Nurdianto AR (2021). A mixture of ceramic biomaterials (hydroxyapatite and β-tricalcium phosphate) and chitosan as a scaffold for critical sized defect bone. Conserv Dent J.

[9] Xidaki D, Agrafioti P, Diomatari D (2018). Synthesis of hydroxyapatite, β-tricalcium phosphate and biphasic calcium phosphate particles to act as local delivery carriers of curcumin: loading, release and in vitro studies. Materials (Basel).

[10] Kharissova OV, Méndez YP, Kharisov BI, Nikolaev AL, Dorozhkin SV, Mena DN, García BO (2025). Biomineralization of calcium phosphates in nature. Nano-Struct Nano-Objects.

[11] Webler GD, Zapata MJM, Agra LC, Barreto E, Silva AOS, Hickmann JM, Fonseca EJS (2014). Characterization and evaluation of cytotoxicity of biphasic calcium phosphate synthesized by a solid state reaction route. Curr Appl Phys.

[12] Cho JS, Ko YN, Koo HY, Kang YC (2010). Synthesis of nano-sized biphasic calcium phosphate ceramics with spherical shape by flame spray pyrolysis. J Mater Sci Mater Med.

[13] Chen J, Wang Y, Chen X, Ren Li, Lai Chen, He Wen, Qiqing Z (2011). A simple sol-gel technique for synthesis of nanostructured hydroxyapatite, tricalcium phosphate and biphasic powders. Mater Lett.

[14] Pena J, Vallet-Regı M (2003). Hydroxyapatite, tricalcium phosphate and biphasic materials prepared by a liquid mix technique. J Eur Ceram Soc.

[15] Sopyan I, Natasha AN (2009). Preparation of nanostructured manganese-doped biphasic calcium phosphate powders via sol-gel method. Ionics.

[16] Natasha AN, Singh R, Bin Abd Shukor MH, Tan CY, Purbolaksono J, Sopyan I, Toulouei R (2014). Synthesis and properties of biphasic calcium phosphate prepared by different methods. Adv Mater Res.

[17] Gibson IR, Rehman I, Best SM, Bonfield W (2000). Characterization of the transformation from calcium-deficient apatite to beta-tricalcium phosphate. J Mater Sci Mater Med.

[18] Topsakal A, Ekren N, Kilic O (2020). Synthesis and characterization of antibacterial drug loaded β-tricalcium phosphate powders for bone engineering applications. J Mater Sci Mater Med.

[19] Tran DL, Ta QT, Tran MH (2025). Optimized synthesis of biphasic calcium phosphate: enhancing bone regeneration with a tailored β-tricalcium phosphate/hydroxyapatite ratio. Biomater Sci.

[20] Massit A, Fathi M, El Yacoubi A, Kholtei A, El Idrissi BC (2018). Effect of physical and chemical parameters on the β-tricalcium phosphate synthesized by the wet chemical method. Mediterr J Chem.

[21] Othman R, Mustafa Z, Kien PT, Ishak NF, Shaaban A, Noor AM (2017). Parameters affecting the synthesis of β-tricalcium phosphate powder using a wet precipitation method. J Mech Eng Sci.

[22] Yoshida K, Kobayashi M, Hyuga H, Kondo N, Kita H, Hashimoto K, Toda Y (2007). Reaction sintering of β-tricalcium phosphates and their mechanical properties. J. Eur. Ceram. Soc.

[23] Brazete D, Torres PMC, Abrantes JC, Ferreira JM (2018). Influence of the Ca/P ratio and cooling rate on the allotropic α↔β-tricalcium phosphate phase transformations. Ceram Int.

[24] Mirhadi B, Mehdikhani B, Askari N (2011). Synthesis of nano-sized β-tricalcium phosphate via wet precipitation. Process Appl Ceram.

[25] Kwon S, Jun Y, Hong S, Kim H (2003). Synthesis and dissolution behavior of b-TCP and HA/b-TCP composite powders. J Eur Ceram Soc.

[26] Sych EE, Pinchuk ND, Tovstonog AB (2014). The structure and properties of calcium phosphate ceramics produced from monetite and biogenic hydroxyapatite. Powder Metall Met Ceram.

[27] Sych OY, Pinchuk ND, Pasichnyi VV, Ostapenko SO, Kotlyarchuk AV, Tovstonog GB, Yevich YI (2015). Structure and properties of ceramics based on monetite and nanodispersed silica. Powder Metall Met Ceram.

[28] Safronova TV, Putlyaev VI, Shekhirev MA, Kuznetsov AV (2007). Composite ceramic containing a bioresorbable phase. Glass Ceram.

[29] Koenig ZA, Burns JC, Hayes JD (2022). Necrotic granulomatous inflammation after use of small intestine submucosa matrix for recurrent compression neuropathy. Plast Reconstr Surg Glob Open.

